# A local QSAR model based on the stability of nitrenium ions to support the ICH M7 expert review on the mutagenicity of primary aromatic amines

**DOI:** 10.1186/s41021-022-00238-1

**Published:** 2022-03-21

**Authors:** Ayaka Furukawa, Satoshi Ono, Katsuya Yamada, Nao Torimoto, Mahoko Asayama, Shigeharu Muto

**Affiliations:** 1 Safety Research Laboratories, Innovative Research Division, Mitsubishi Tanabe Pharma Corporation, 26-1, Muraoka-Higashi 2-chome, Fujisawa, Kanagawa 251-8555 Japan; 2 Discovery Technology Laboratories, Innovative Research Division, Mitsubishi Tanabe Pharma Corporation, 1000, Kamoshida-cho, Aoba-ku, Yokohama, Kanagawa 227-0033 Japan

**Keywords:** Primary aromatic amine, Quantitative structure–activity relationship (QSAR), In silico, Nitrenium ion, ICH M7, Expert review

## Abstract

**Background:**

Aromatic amines, often used as intermediates for pharmaceutical synthesis, may be mutagenic and therefore pose a challenge as metabolites or impurities in drug development. However, predicting the mutagenicity of aromatic amines using commercially available, quantitative structure–activity relationship (QSAR) tools is difficult and often requires expert review. In this study, we developed a shareable QSAR tool based on nitrenium ion stability.

**Results:**

The evaluation using in-house aromatic amine intermediates revealed that our model has prediction accuracy of aromatic amine mutagenicity comparable to that of commercial QSAR tools. The effect of changing the number and position of substituents on the mutagenicity of aromatic amines was successfully explained by the change in the nitrenium ion stability. Furthermore, case studies showed that our QSAR tool can support the expert review with quantitative indicators.

**Conclusions:**

This local QSAR tool will be useful as a quantitative support tool to explain the substituent effects on the mutagenicity of primary aromatic amines. By further refinement through method sharing and standardization, our tool can support the International Council for Harmonisation of Technical Requirements for Pharmaceuticals for Human Use (ICH) M7 expert review with quantitative indicators.

## Introduction

The International Council for Harmonisation of Technical Requirements for Pharmaceuticals for Human Use (ICH) M7 guideline describes the process of hazard identification and risk assessment of impurities that may be present in a drug substance or product [1]. Risk assessment allows the assessment of mutagenicity using quantitative structure–activity relationship (QSAR) tools, which enables the screening of numerous potential impurities. ICH M7 recommends that bacterial mutagenicity should be assessed using two QSAR methodologies, namely, by expert rule-based and statistics-based methodologies. The prediction accuracy of QSAR tools is improved by increasing the number of training sets and modifying algorithms [2, 3]. However, the mutagenicity of several structures, such as aromatic amines, is still difficult to predict when using QSAR tools [4, 5].

Because aromatic amines are widely used as intermediates in pharmaceutical synthesis and can remain as impurities, their mutagenicity is a serious safety issue [4, 5]. In addition, aromatic amines can be formed as metabolites, especially for drugs with amide bonds that may easily break down by enzymatic hydrolysis [6, 7].

Predicting the mutagenicity of aromatic amines remains a major challenge for QSAR tools because positive predictions are sometimes false positives. Therefore, an improvement of prediction accuracy is strongly desired from the viewpoint of regulatory science.

In recent years, several efforts have been made to improve the prediction accuracy of QSAR tools. One method is to share nonpublic data held by pharmaceutical companies, as existing QSAR tools have been built based on public knowledge [4, 5]. Another method is to perform expert reviews. The SAR fingerprint, a chemical fingerprint of aromatic amine mutagenicity developed by Ahlberg et al. [4], is a useful approach to performing expert reviews [4, 8–10]. However, the SAR fingerprint approach is difficult to apply to mutagenicity assessment when activating and deactivating substituents are simultaneously present. We considered whether quantitative predictive indices based on mutagenic mechanisms can complement the SAR fingerprinting approach.

**Fig. 1:**
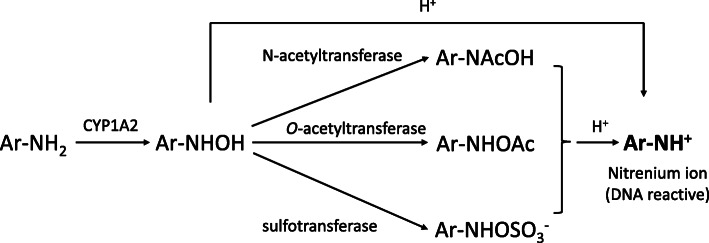
Mechanism of mutagenicity induced by aromatic amines

The mechanism underlying mutagenic induction of aromatic amines has been well studied (Fig. 1). In the metabolic activation of aromatic amines, first, hydroxylamine is generated by *N*-hydroxylation by cytochrome P450 enzymes (CYPs; mainly CYP1A2). The hydroxylamine is either conjugated by phase II enzymes (*O*-acetyltransferases, *N*-acetyltransferases, or sulfotransferases) or directly hydrolyzed to nitrenium ions, which form covalent bonds with DNA [11]. Several local QSAR tools have been evaluated in terms of ease of hydroxylamine formation [12, 13], ease of nitrenium ion formation [14], and nitrenium ion stability [15–17].

These local QSAR tools are useful, but they are not user friendly for genotoxicologists. Therefore, this study developed a local QSAR model based on nitrenium ion stability that would be easy to use for genotoxicologists and would support ICH M7 expert reviews with quantitative indicators.

## Materials and methods

### Data set

Two data sets were used in this study, namely, an in-house primary aromatic amine data set used for evaluating QSAR tool performance and a data set of 23 known primary aromatic amines. The in-house data set contained 85 aromatic amines, of which 51 were mutagenic, and 34 were nonmutagenic, and they were collected using the following procedure. Primary aromatic amines with standard Ames [18], fluctuation Ames [19], or Vitotox [20] test results were extracted from the in-house compound database. The aromatic amines were classified as follows: mutagenic (clearly positive under any condition in any of the tests) and nonmutagenic (negative in at least three strains (TA98, TA100, TA1537 [or TA2637]) in the standard Ames test (in the absence and presence of S9). We further narrowed down the aromatic amines using six criteria, as described by Bentzien et al. [16]: (i) no formal charge, (ii) molecular weight of < 500 Da, (iii) maximum one stereocenter, (iv) < 10 rotatable bonds, (v) only one aromatic amine functionality, and (vi) no aromatic nitro groups.

The data set of known primary aromatic amines consisted of the following structures: a) aniline as a standard, b) aniline with methyl groups (7 structures), c) aniline with a methoxy group (3 structures), d) aniline with a sulfonate group (3 structures), e) aniline with a sulfonamide group (2 structures), and f) primary aromatic amines for which expert reviews have been reported [4, 8, 9] and their analogs (7 structures).

### Commercial QSAR tools

In this study, the following commercial mutagenicity prediction QSAR tools were used: Derek Nexus, version 6.1.0, 2020.1.0 version of the knowledge base (Lhasa Limited, UK); CASE Ultra rule-based, version 1.8.0.5, GT_EXPERT v1.8.0.1.16392.500 (MultiCASE Inc., USA); CASE Ultra statistical based, version 1.8.0.5, GT1_BMUT v1.8.0.1.11479.500 (MultiCASE Inc., USA); and Leadscope Model Applier, version 3.0.1–1, Bacterial Mutation v2 (Leadscope Inc., USA).

### Calculating the stability of nitrenium ions

Bentzien et al. reported an in silico method of predicting the Ames mutagenicity of primary aromatic amines using the nitrenium ion hypothesis of Ford et al. [15, 16]. The mutagenic effect of primary aromatic amines is described as the formation of a reactive nitrenium ion, and the authors concluded that nitrenium ion stability correlates with the mutagenic potential. They calculated nitrenium ion stability using a semi-empirical quantum mechanical method and the relative energy ΔΔE:

$$ \Delta  \Delta  E=\Delta  {E}_{ArNH^{+}}+\Delta  {E}_{PhNH_2}-\Delta  {E}_{ArNH_2}-\Delta  {E}_{PhNH^{+}} $$

We used the heat of formation energies of Austin model 1 (AM1) [21] optimized structures calculated using MOPAC v7.1 bundled with Molecular Operation Environment (MOE) 2019.01 software (Chemical Computing Group ULC, Canada). To calculate ΔΔE, we first prepared a structure-data file of the molecule and constructed a 3D structure using the “rebuild 3D” feature of MOE. Then, we designed and performed conformational sampling to accurately calculate the conformational state using LowModeMD [22] with a force field (e.g., MMFF94x) for each neutral molecule within an energy cutoff of 7 kcal/mol. Subsequently, we performed geometry optimization using semi-empirical quantum mechanical calculations with AM1 Hamiltonian for each neutral conformer. The most stable conformer (i.e., with the lowest heat of formation) was selected to determine the nitrenium ion species, one of the amine hydrogens was replaced by a dummy atom X, and geometry optimization was again performed using the keyword CHARGE = +1. The lowest ΔΔE value was adopted for this molecule. The ΔΔE of aniline was set to 0 kcal/mol. If the geometry optimization was not converged, a not-a-number (NaN) was assigned for that formula. A compound with a negative ΔΔE was predicted to be mutagenic, and a compound with a positive ΔΔE was predicted to be nonmutagenic. The main output file was in csv format; therefore, we could easily use spreadsheet programs. The csv file also had a remark column for errors, for example, a NaN, an aromatic ring opening during the geometry optimization, or no aromatic amine.

This procedure was written in Scientific Vector Language (SVL), which is integrated in the MOE modeling package and is freely available to MOE users from MOLSIS Inc. upon request.

### Analysis of QSAR tool performance

To evaluate the predictive performance of the QSAR model, we used compounds that are not included in known databases or commercial QSAR training sets. Therefore, the performance of the QSAR tools was evaluated using in-house aromatic amine data only. The performance metrics employed were accuracy, sensitivity, specificity, positive prediction value, negative prediction value, Matthews correlation coefficient (MCC), and coverage [23].

## Results

### Analysis of QSAR tool performance using in-house aromatic amines

**Fig. 2:**
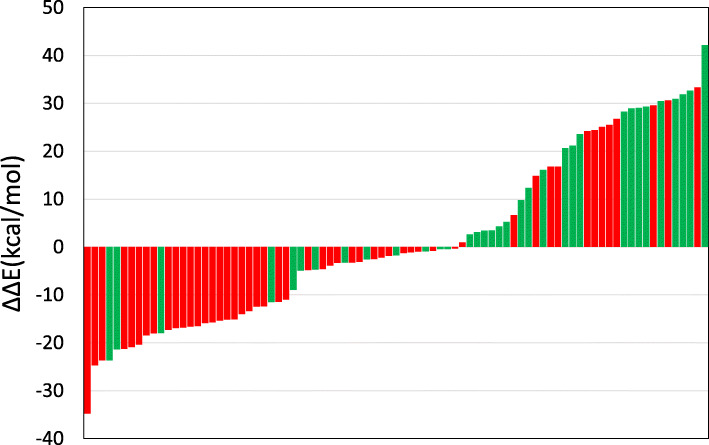
Mutagenicity and ΔΔE distribution of in-house primary aromatic amines. Each bar indicates a compound. Mutagens are shown in red and nonmutagens in green. The aromatic amines are arranged in order of their ΔΔE values (kcal/mol)

Figure 2 shows the ΔΔE of each in-house aromatic amine and Ames test results. The ΔΔE values were small for mutagens and large for nonmutagens. Table 1 shows the predictive performance of commercial QSAR tools and ΔΔE against the in-house aromatic amines.

**Table 1:** Comparison of the predictive performance of QSAR tools for in-house aromatic amines

	Derek Nexus	GT_EXPERT	GT1_BMUT	Leadscope Model Applier	ΔΔE
Accuracy	57.6%	44.7%	57.6%	50.0%	69.4%
Sensitivity	35.3%	39.2%	62.5%	43.2%	74.5%
Specificity	91.2%	52.9%	50.0%	61.5%	61.8%
Positive prediction value	85.7%	55.6%	65.8%	65.5%	74.5%
Negative prediction value	48.4%	36.7%	46.4%	39.0%	61.8%
Matthews correlation coefficient	0.3	−0.1	0.1	0.0	0.4
Coverage	100%	100%	77.6%	82.3%	100%
Inconclusive	N/A	N/A	22.4%	10.6%	N/A
Out of domain	N/A	N/A	N/A	7.1%	N/A

*QSAR* Quantitative structure–activity relationship, *N/A* Not applicable

The prediction accuracy of our in-house aromatic amines using ΔΔE was about 70%, which is comparable to the reported prediction accuracy of commercial QSAR tools for aromatic primary amines. The prediction accuracies of commercial QSAR tools for primary aromatic amines are 59 to 64% for 599 test compounds and can be improved to about 63 to 79% by adding new data sets and revising the rules [4]. For another 268 test compounds, the reported accuracy ranged from 57 to 73% [5].

The prediction accuracies of commercially available QSAR tools for in-house developed aromatic amines were about 45–58%, which are slightly lower than the reported value. The performance of commercial QSAR tools depends on the quantity, quality, and diversity of the training set. Therefore, we considered the possibility of improving the accuracy by training with our proprietary compounds. On the other hand, since the ΔΔE calculation lacks the concept of learning by structure sets, predictions for new structures are not expected to affect the accuracy.

### “Quantitative” SAR fingerprint approach

**Fig. 3:**
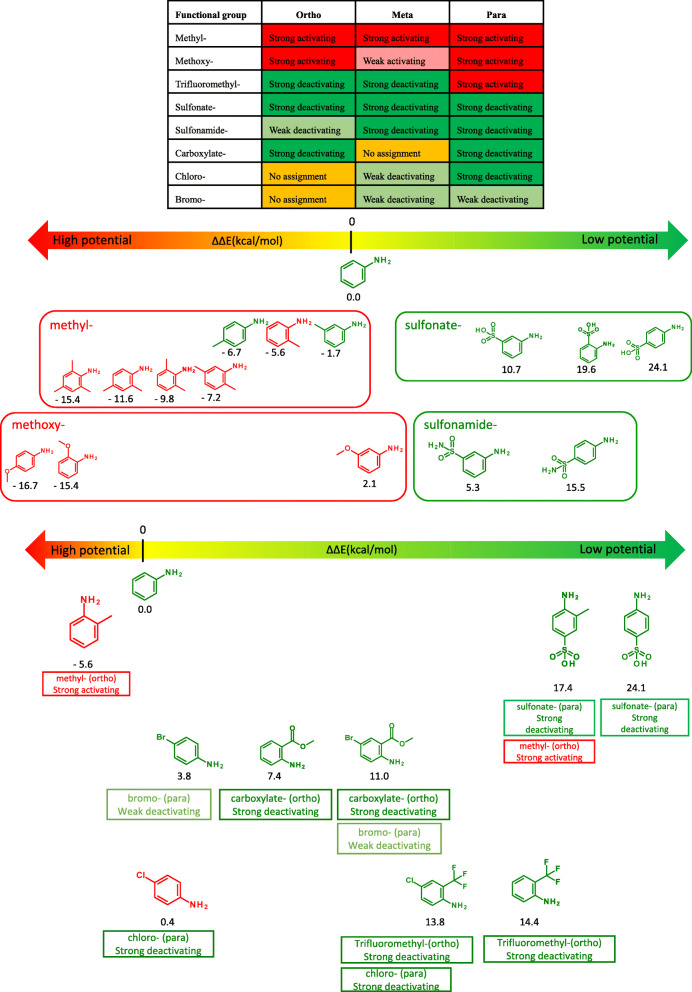
Effect of activating and deactivating substituents on ΔΔE and mutagenicity of aromatic amines. The structures of mutagens are shown in red and those of nonmutagens in green. The numbers below the structures indicate ΔΔE values. The text in the squares shows the classification by Ahlberg et al

Figures 3 show the results of quantitative chemical fingerprinting (termed “SAR fingerprint”) of aromatic amines for the representative substituents. According to Ahlberg et al. [4], methyl groups in ortho-, meta-, and para-positions are activating substituents. One methyl group each in the ortho-, meta-, and para-positions decreased ΔΔE. The ΔΔE was lower for aromatic amines with two or three methyl groups, which tested positive (mutagenic) in the Ames test. For the methoxy group, which is an activating substituent, the ΔΔE decreased, except when the methoxy group was in the meta-position, and the aromatic amines with these groups tested positive in the Ames test. Sulfonate and sulfonamide groups, which are considered deactivating substituents, increased the ΔΔE in the ortho-, meta-, and para-positions, and aromatic amines with these groups tested negative in the Ames test.

### ICH M7 expert review case studies

The applicability of the ΔΔE method was examined for several compounds for which expert reviews based on ICH M7 have been reported (Table 2).

**Table 2:** Aromatic amines used in the “Quantitative” SAR fingerprint approach

No.	Structure	CAS number	Ames	ΔΔE (kcal/mol)	GT1_BMUT	Leadscope	Derek	GT_Expert	Ref.
1		142-04-1	Non mutagen	0	Inconclusive	Indeterminate	Inactive	Known Negative	[24]
2		95-53-4	Mutagen	–5.6	Positive	Indeterminate	Inactive	Known Positive	[25]
3		108-44-1	Non mutagen	–1.7	Inconclusive	Indeterminate	Inactive	Known Negative	[26]
4		106-49-0	Non mutagen	–6.7	Positive	Active	Inactive	Known Negative	[26]
5		87-62-7	Mutagen	–9.8	Negative	Indeterminate	Plausible	Known Positive	[27]
6		95-68-1	Mutagen	–11.6	Positive	Active	Plausible	Known Positive	[27]
7		95-78-3	Mutagen	–7.2	Positive	Indeterminate	Plausible	Known Positive	[27]
8		88-05-1	Mutagen	–15.4	Inconclusive	Active	Plausible	Known Positive	[28]
9		134-29-2	Mutagen	–15.4	Positive	Active	Plausible	Known Positive	[29]
10		536-90-3	Mutagen	2.1	Positive	Inactive	Plausible	Known Positive	[30]
11		104-94-9	Mutagen	–16.7	Positive	Active	Plausible	Known Positive	[30]
12		88-21-1	Non mutagen	19.6	Positive	Inactive	Inactive	Known Negative	[31]
13		121-47-1	Non mutagen	10.7	Negative	Inactive	Inactive	Known Negative	[27]
14		121-57-3	Non mutagen	24.1	Negative	Inactive	Inactive	Known Negative	[27]
15		98-18-0	Non mutagen	5.3	Negative	Inactive	Inactive	Negative	[5]
16		63-74-1	Non mutagen	15.5	Negative	Inactive	Inactive	Known Negative	[32]
17		98-33-9	Non mutagen	17.4	Positive	Inactive	Inactive	Known Negative	[4, 33]
18		52727-57-8	Non mutagen	11	Negative	Inactive	Inactive	Known Negative	[8, 34]
19		445-03-4	Non mutagen	13.8	Negative	Inactive	Inactive	Known Negative	[9]
20		106-40-1	Non mutagen	3.8	Positive	Inactive	Inactive	Known Negative	[27]
21		134-20-3	Non mutagen	7.4	Negative	Inactive	Inactive	Known Negative	[35]
22		88-17-5	Non mutagen	14.4	Negative	Inactive	Inactive	Known Negative	[36]
23		106-47-8	Mutagen	0.4	Positive	Inactive	Inactive	Known Positive	[29]

#### Case study 1

4-Amino-3-methylbenzenesulfonic acid, which is No. 17 in Table 2, is compound D in the report by Ahlberg et al. [4]. Given that this compound has strong activating (methyl in the ortho-position) and deactivating (sulfonate in the para-position) substituents simultaneously, we opted to use it for quantitative evaluation. In addition, their paper did not specify the results predicted by commercial QSAR tools for this compound, and our study showed conflicting results with those from commercial QSAR tools (Table 2).

The methyl group in the ortho-position slightly decreased the ΔΔE, whereas the sulfonate group in the para-position caused a significant increase. The ΔΔE of this compound was approximately the sum of the ΔΔE values of aromatic amines with a methyl group in the ortho-position and a sulfonic acid group in the para-position, and it was predicted to be nonmutagenic and tested negative in the Ames test (Fig. 3, Table 2) [33]. The results suggest that the quantitative index complements the SAR fingerprint approach of aromatic amine mutagenicity developed by Ahlberg et al. [4].

#### Case study 2

Methyl 2-amino-5-bromobenzoate, which is No. 18 in Table 2, is example 10 in the expert review by Amberg et al. [8]. We used this compound as a case study because their paper reported that commercial QSAR tools give conflicting results. This compound is reported to be nonmutagenic by the expert rule-based model and inconclusive by the statistics-based model.

The bromo group in the para-position and carboxylate in the ortho-position increased the ΔΔE, and the compound was predicted to be less mutagenic (Fig. 3, Table 2) and tested negative in the Ames test [34]. The bromo group in the para-position and carboxylate in the ortho-position are reported to be deactivating substituents [4]; thus, the results support the expert review with quantitative indicators.

#### Case study 3

2-Amino-5-chlorobenzotrifluoride, which is No. 19 in Table 2, is case 5 in the report by Mishima et al. [9]. This compound was also used as a case study because their paper reported conflicting results with a commercial QSAR tool. This compound is reported to be nonmutagenic by the expert rule-based model and mutagenic by the statistics-based model.

The chloro group in the para-position slightly increased the ΔΔE, and the compound was reported to be mutagenic. The compound with a trifluoromethyl group in the ortho-position had a large ΔΔE and was nonmutagenic. The ΔΔE of this compound with both substituents was approximately the sum of the ΔΔE values of aromatic amines with a chloro group in the para-position and a trifluoromethyl group in the ortho-position and was predicted to be nonmutagenic and tested negative in the Ames test (Fig. 3, Table 2) [37]. The chloro group in the para-position and carboxylate in the ortho-position are reported to be deactivating substituents [4]; thus, the results also support the expert review with quantitative indicators.

In the known primary aromatic amine data set, several compounds gave conflicting results in the commercial QSAR tools (Table 2). The mutagens, namely compounds No. 5, 7, and 8, were accurately predicted in expert rule-based models but were predicted negative, inconclusive, or indeterminate in statistics-based models. These compounds were predicted to be mutagenic with ΔΔE values of −9.8, −7.2, and −15.4 kcal/mol, respectively, supporting the prediction by expert rule-based models. Compounds No. 12 and 17, which are nonmutagenic, were correctly predicted by the expert rule-based models but were predicted as positive in the statistics-based model. These compounds were predicted to be nonmutagenic with ΔΔE values of 19.6 and 17.4 kcal/mol, respectively, supporting the prediction by the expert rule-based model. Accurate prediction of the mutagenicity of compounds with ΔΔE values in the range of about ±5 kcal/mol was difficult, suggesting that an appropriate cutoff value should be set [16].

## Discussion

Mechanism-based local QSAR tools are useful; however, they are not user friendly for genotoxicologists and have not been standardized. To make such tools widely applicable to safety assessment for industry and regulatory purposes, we developed a shareable procedure in SVL for predicting aromatic amine mutagenicity based on the concept described by Bentzien et al. [16]. The difference is that in our method, ΔΔE is calculated after selecting the most stable conformer by a conformational search using a force field. This difference makes it easier for genotoxicologists to use our method, as they can now perform everything from compound structure preprocessing to ΔΔE calculations using the MOE software.

The evaluation using in-house aromatic amine intermediates showed that our QSAR tool has a mutagenicity prediction accuracy comparable to that of commercial QSAR tools. Given the complex structure of in-house compounds, characterization of all the compounds that were not predicted correctly was difficult. However, the compounds that resulted in false positive predictions had large substituents. The presence of bulky substituents can inhibit the metabolic activation of aromatic amines by CYP1A2 due to steric hindrance. Alternatively, electron-withdrawing substituents can have a resonance effect on aromatic rings, reducing electron density and disrupting the electron distribution necessary for metabolic activation [13]. These factors are not reflected in the calculation of ΔΔE values, which may lead to false positive predictions.

We also investigated the possibility of supporting the SAR fingerprint of aromatic amines [4], which involves a qualitative evaluation. The effect of changing the number and position of substituents on the mutagenicity of aromatic amines is successfully explained by the change in ΔΔE. Furthermore, case studies using several aromatic amines evaluated by the ICH M7 expert review showed that our QSAR tool can support the expert review with quantitative indicators. In particular, when activating and deactivating substituents coexist, as in case study 1, it is difficult to predict mutagenicity by qualitative evaluation; however, we believe that our method can provide important information.

Using in-house compound data set, we examined the cutoff value of ΔΔE in increments of 2.5 kcal/mol between −10 and +10 kcal/mol and found that accuracy and MMC were the highest when the cutoff value was based on the value of +2.5 kcal/mol (Table 3). The cutoff value may change depending on the validation compound used, and further investigation using more compounds is necessary.

**Table 3:** Examination of the cutoff value of ΔΔE using in-house compound data set

Cutoff value (kcal/mol)	−10.0	−7.5	−5.0	−2.5	0	+2.5	+5.0	+7.5	+10.0
Accuracy	63.5%	62.4%	62.4%	65.9%	69.4%	70.6%	64.7%	64.7%	63.5%
Sensitivity	85.7%	82.8%	82.8%	77.5%	74.5%	75.0%	68.4%	67.8%	66.7%
Specificity	52.6%	51.8%	51.8%	55.6%	61.8%	63.6%	57.1%	57.7%	56.0%
Positive prediction value	47.1%	47.1%	47.1%	60.8%	74.5%	76.5%	76.5%	78.4%	78.4%
Negative prediction value	88.2%	85.3%	85.3%	73.5%	61.8%	61.8%	47.1%	44.1%	41.2%
Matthews correlation coefficient	−0.2	−0.2	−0.2	−0.2	0.4	0.4	0.2	0.2	0.2
Coverage	100%	100%	100%	100%	100%	100%	100%	100%	100%
Inconclusive	N/A	N/A	N/A	N/A	N/A	N/A	N/A	N/A	N/A
Out of domain	N/A	N/A	N/A	N/A	N/A	N/A	N/A	N/A	N/A

*N/A* Not applicable

In this paper, we have shown the usefulness of the model focusing on aromatic amines. However, in the mutagenic mechanism of aromatic nitros, the nitro group is reduced by nitroreductase to produce nitrenium ions [38]. Although the potential of our method for the application to aromatic nitros has not been investigated, our method can be possibly applied.

This local QSAR model is different from the two complementary QSARs (expert rule-based and statistics-based) recommended in ICH M7. ICH M7 recommends that analyses using these QSAR models should be performed first, and expert review should be considered when necessary. We hope that this local QSAR model is useful in making decisions during expert reviews.

Further validation using more known and undisclosed aromatic amines may be necessary to clarify the applicability of our method. To facilitate its standardization, the SVL script for the method is provided free of charge to MOE users by MOLSIS Inc. We hope that further refinement of this method will contribute to the standardization of expert reviews on the prediction of mutagenicity of aromatic amines under the ICH M7 guideline.

## Conclusions

A shareable MOE SVL script was developed for predicting the mutagenicity of primary aromatic amines based on nitrenium ion stability. This local QSAR tool will be useful as a quantitative support tool to explain the substituent effects on the mutagenicity of primary aromatic amines. By further refinement, this tool can support the ICH M7 expert review with quantitative indicators.

## Data Availability

All data generated or analyzed during this study are included in this published article.

## Abbreviations

ICH: International Council for Harmonisation of Technical Requirements for Pharmaceuticals for Human Use
MOE: Molecular Operation Environment
AM1: Austin model 1
QSAR: Quantitative structure–activity relationship
SAR: structure–activity relationship
SVL: Scientific Vector Language
MCC: Matthews correlation coefficient
CAS: Chemical Abstracts Service
NaN: Not-a-number
